# Supplementary material for: Expression of CD44v6-Containing Isoforms Influences Cisplatin Response in Gastric Cancer Cells
Source: Cancers (Basel). 2020 Apr 2;12(4):858. doi: 10.3390/cancers12040858 (PMC7226224; doi:10.3390/cancers12040858)
Supplement: Supplementary file 1 [file cancers-12-00858-s001.pdf]

Supplementary Material

Expression of CD44v6 Containing Isoforms Influences Cisplatin Response in Gastric Cancer Cells

Carla Pereira, Daniel Ferreira, Nuno Mendes, Pedro L. Granja, Gabriela M. Almeida and Carla Oliveira

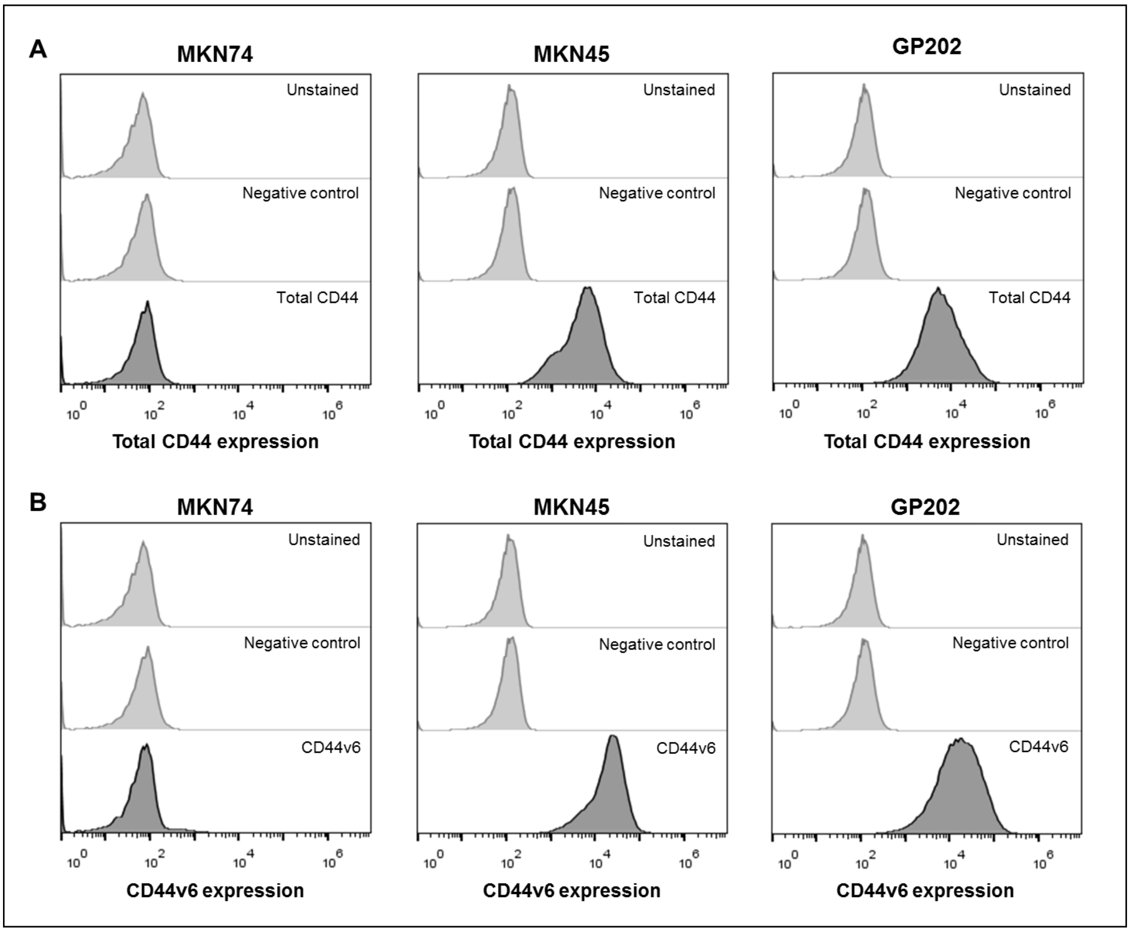

Figure S1. Confirmation that MKN74 cell line is CD44-null and does not express CD44, as determined by flow cytometry. (*A*) No expression of the standard version of total CD44 is detected in MKN74 cells (MKN45 and GP202 cells were used as a positive control for the expression of total CD44); (*B*) No expression of CD44v6 is detected in MKN74 cells (MKN45 and GP202 cells were used as a positive control for CD44v6 expression).

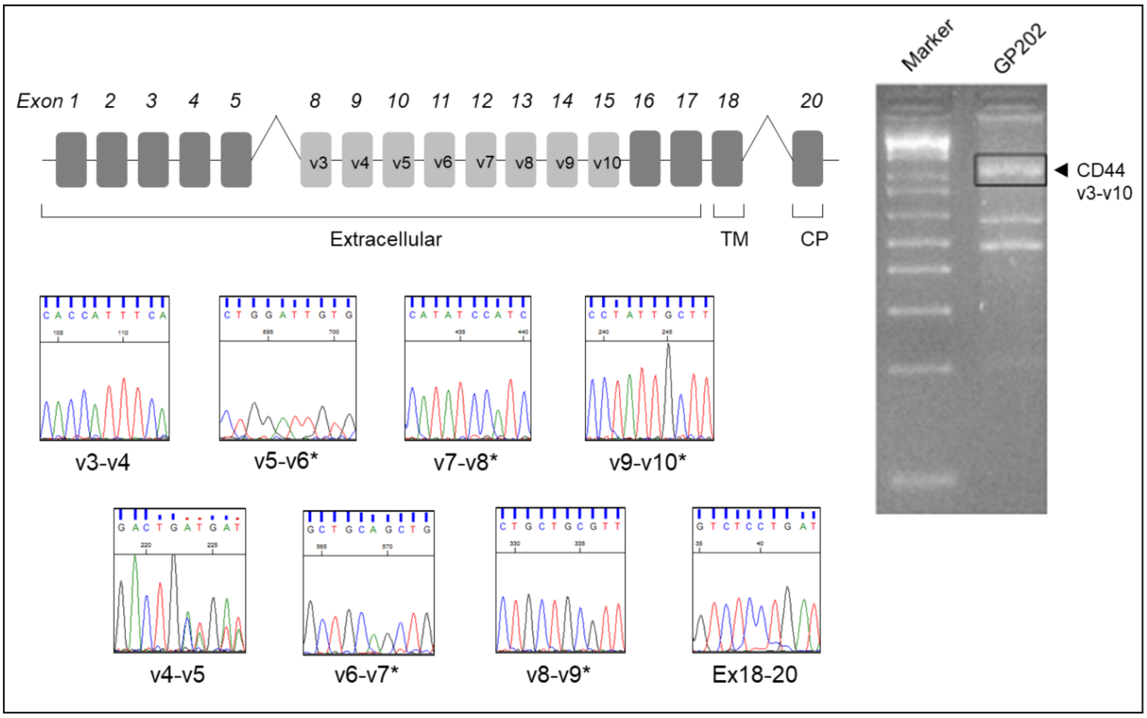

Figure S2. Identification of CD44v3-v10 variant endogenously expressed in the GP202 cell line. The band outlined in the gel represents the CD44v3-v10 transcript. Identity of the transcript was confirmed by Sanger sequencing. Each exon-exon junction in the boundary region was positively identified. It should be noted that the v4-v5 exon boundary displays a double reading frame after the Exonv5 start. One of the sequences has a CAG insertion identified using the mutation surveyor software. *These sequences were obtained with a reverse primer and are shown as the reverse complement of the forward strand.

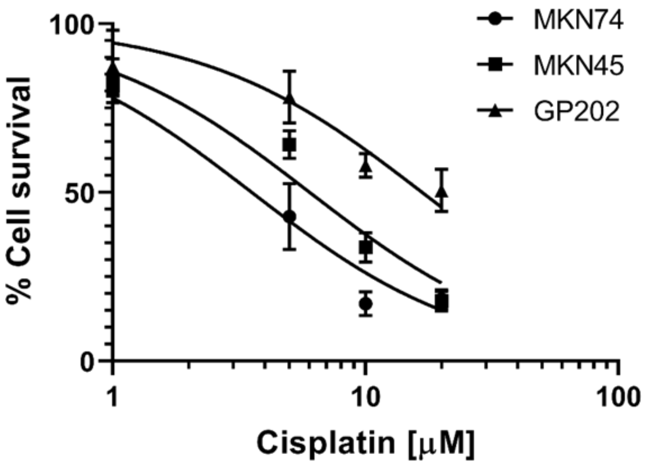

Figure S3. Determination of IC50 values in MKN74, MKN45 and GP202 cell lines. Cells were treated for 48 h with various cisplatin concentrations (1, 5, 10 and 20 μM) and cell survival was determined using a resazurin-based assay (PB). IC50 values were determined by non-linear regression analysis, as follows: MKN74 IC50 ~ 3.6 μM; MKN45 IC50 ~ 6 μM and; GP202 IC50 ~ 17 μM.

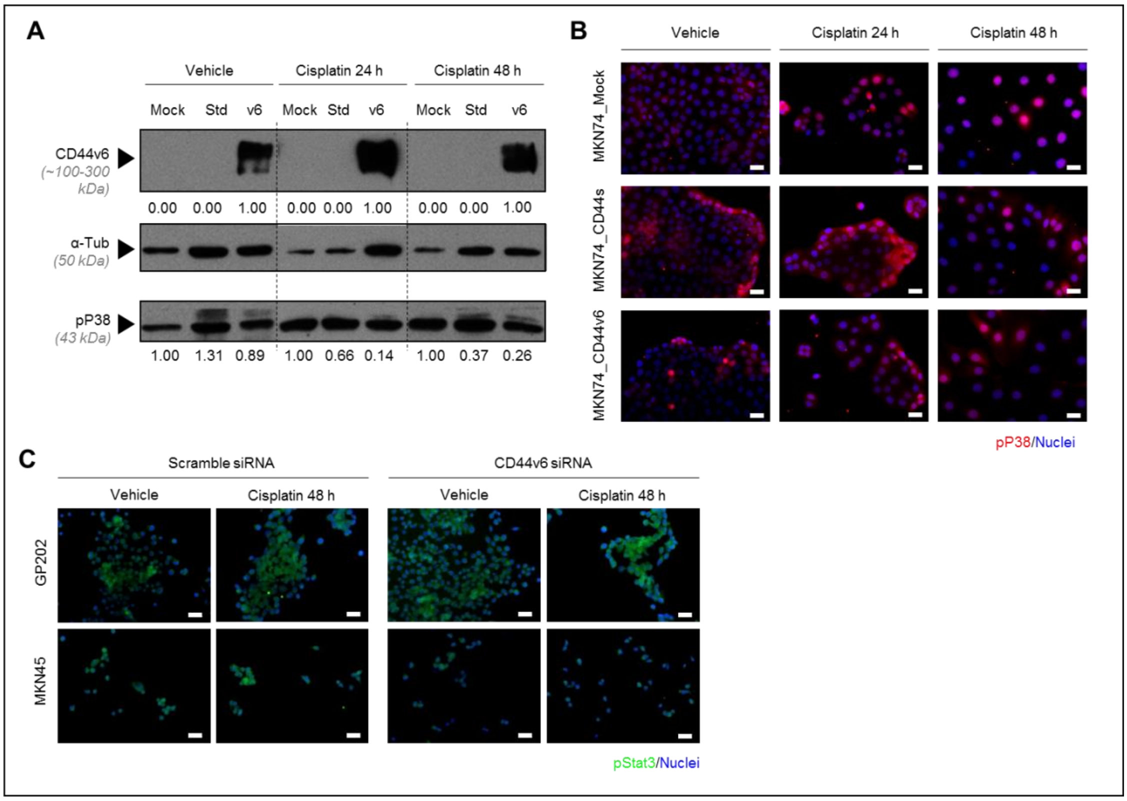

Figure S4. Expression of pP38 and pSTAT3 in MKN74 and GP202/MKN45 cells, respectively. **(A)** Western blotting of pP38 in MKN74 cell lines. CD44v6 and pP38 were run in the same gel as pSTAT3 (shown in Figure 4D) against the same tubulin; **(B)** Immunofluorescence of pP38 (seen in red) in untreated cell lines and upon treatment with cisplatin for 24 and 48 hours; **(C)** Immunofluorescence of pSTAT3 (seen in green) in vehicle and cisplatin treated GP202 and MKN45 cell lines (following a 24 h pre-incubation with scramble or CD44v6 siRNAs). Nuclei are stained with DAPI (seen in blue) and white scale bars represent a distance of 50 µm.

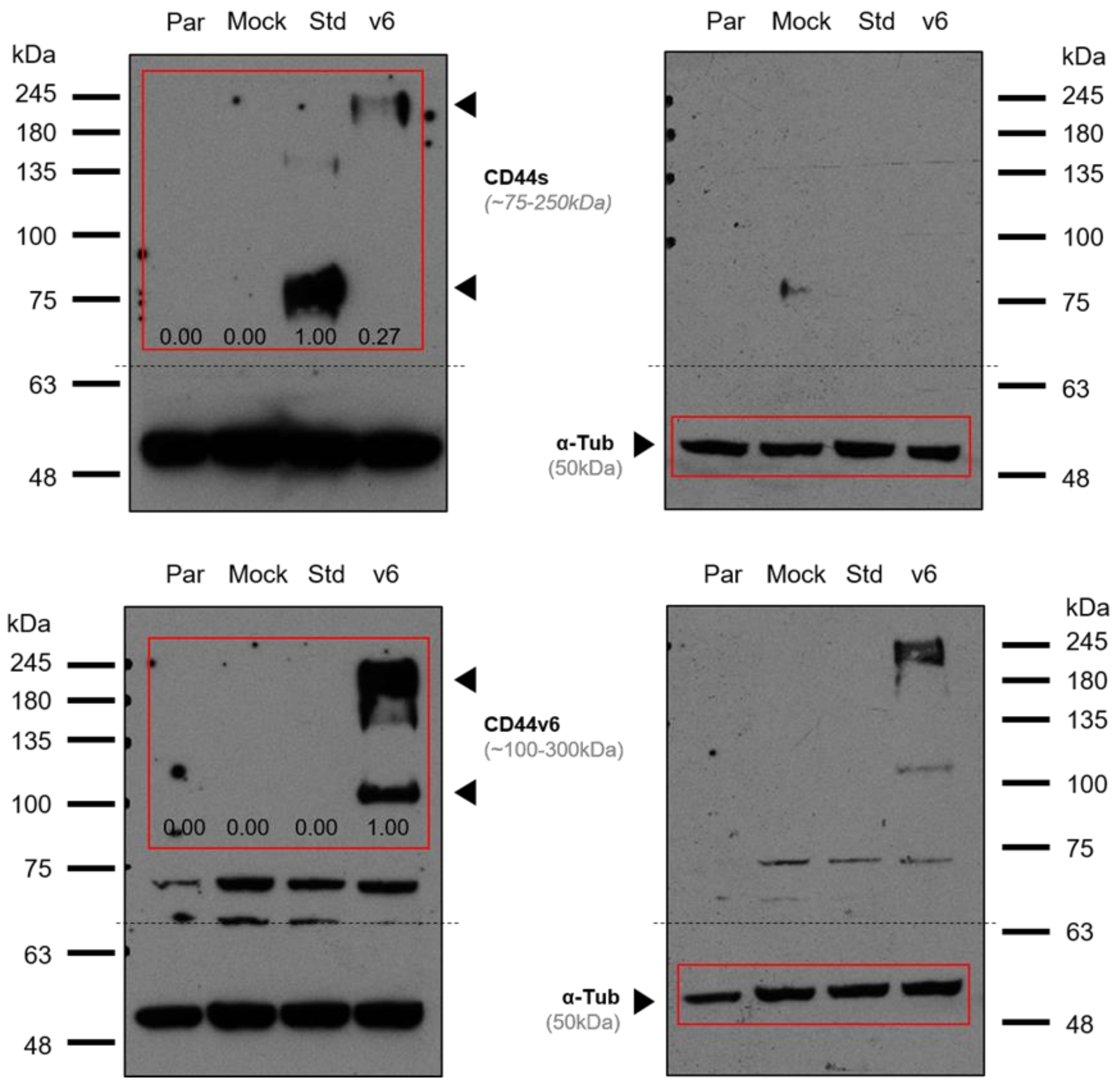

Figure S5. Original Western blots of Figure 1C.

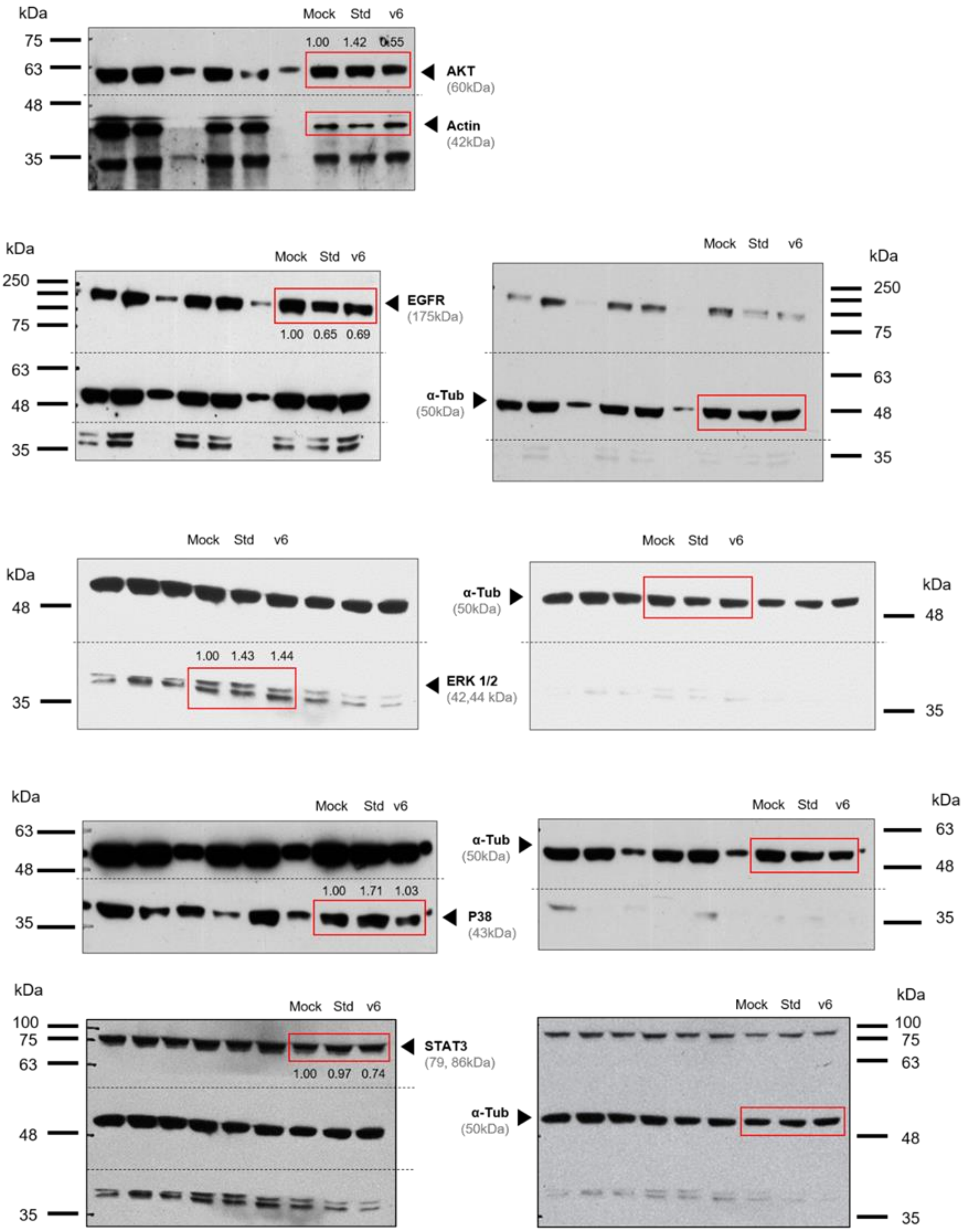

Figure S6. (Part I): Original Western blots of Figure 2E.

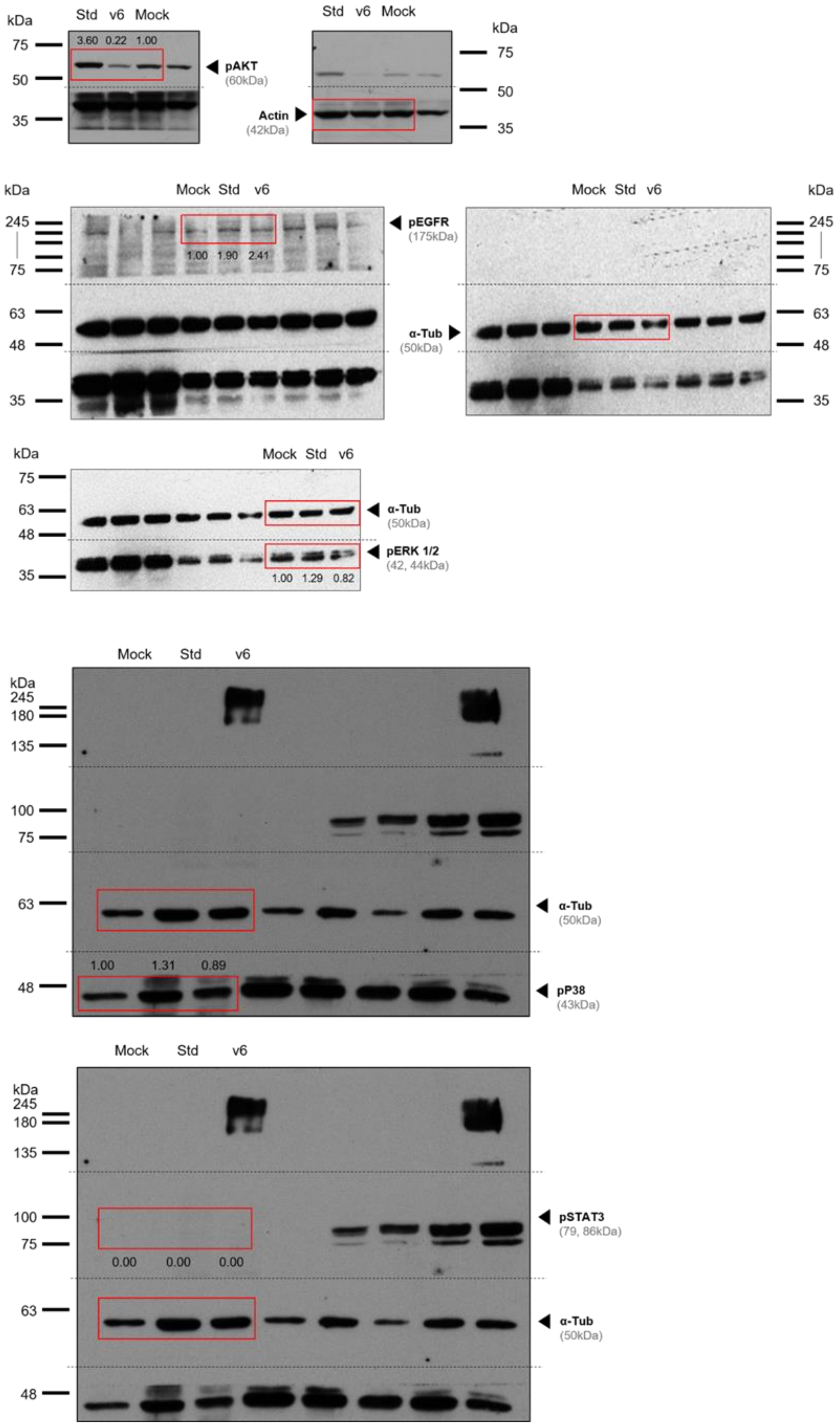

Figure S6. (Part II): Original Western blots of Figure 2E.

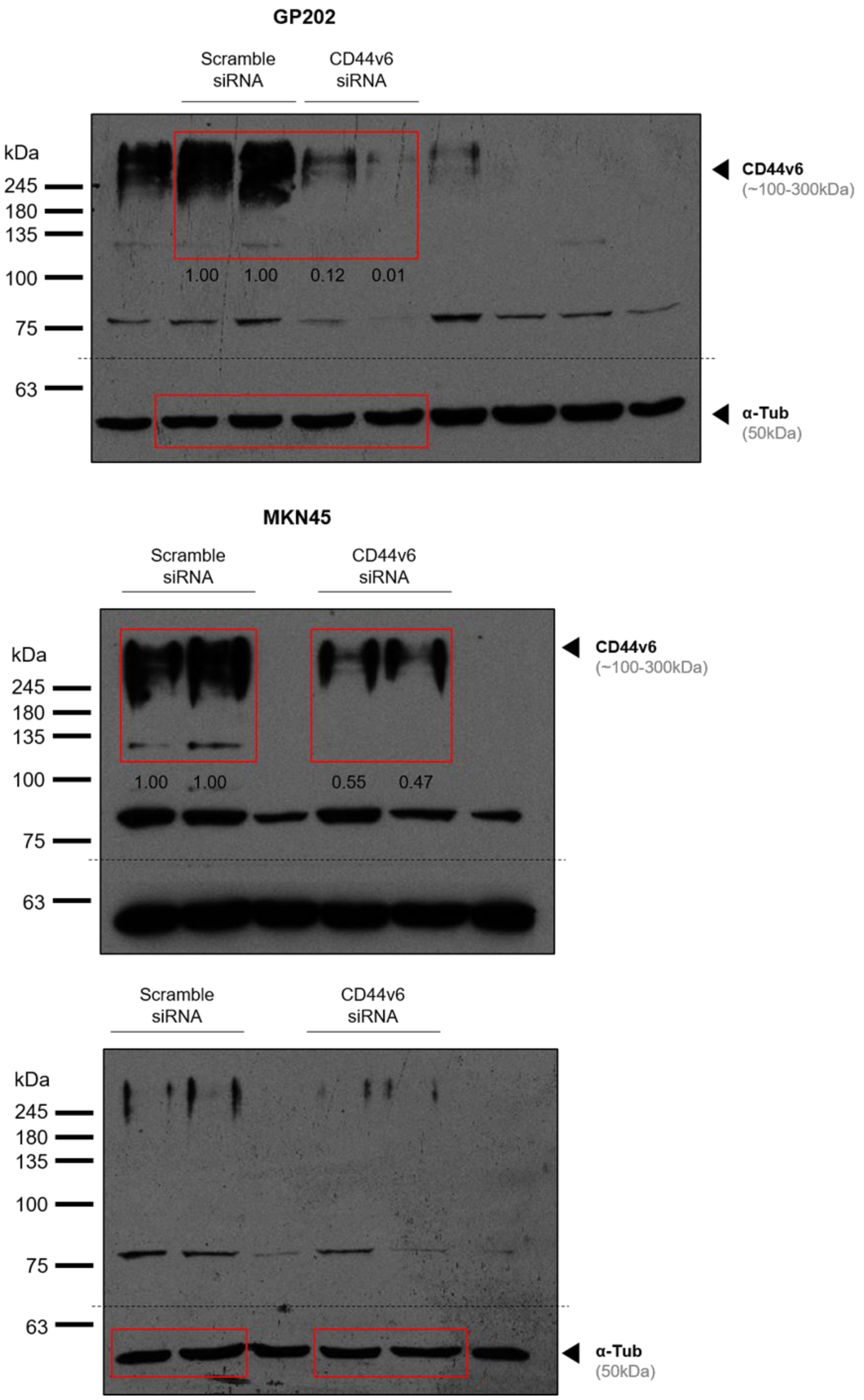

Figure S7. Original Western blots of Figure 4A.

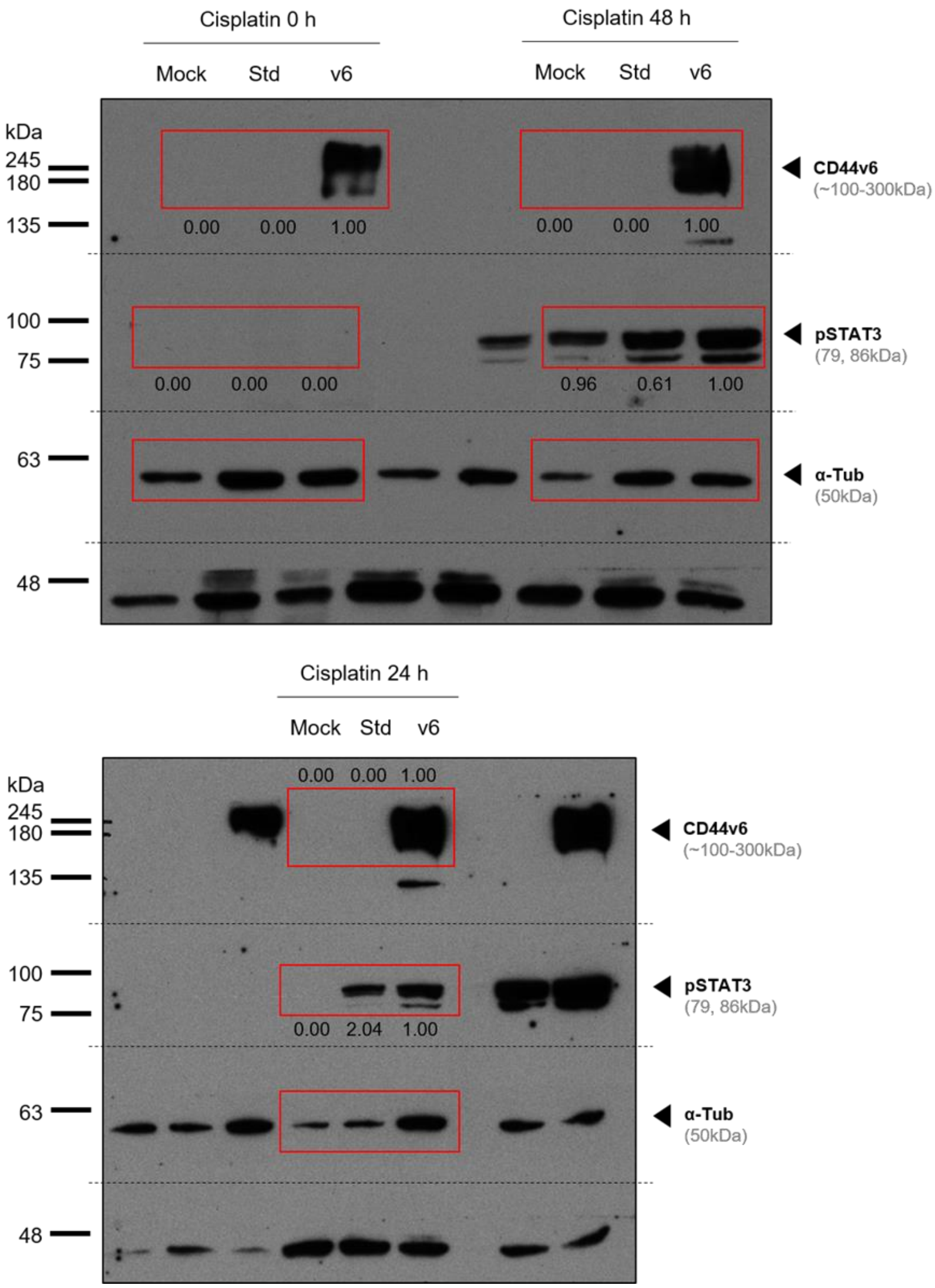

Figure S8. Original Western blots of Figure 4D.

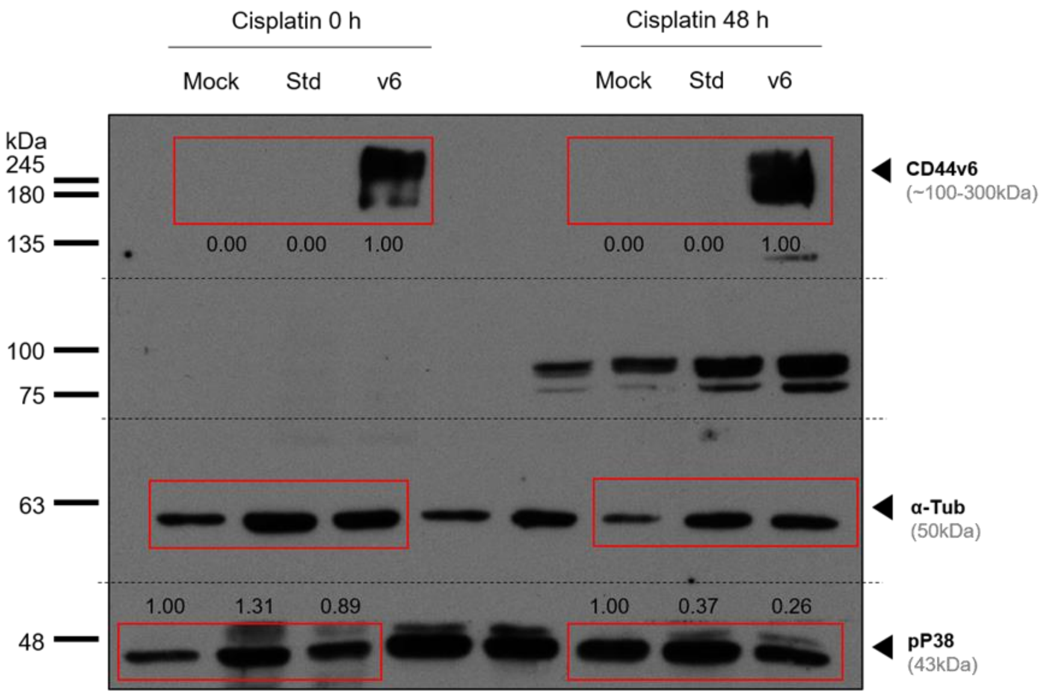

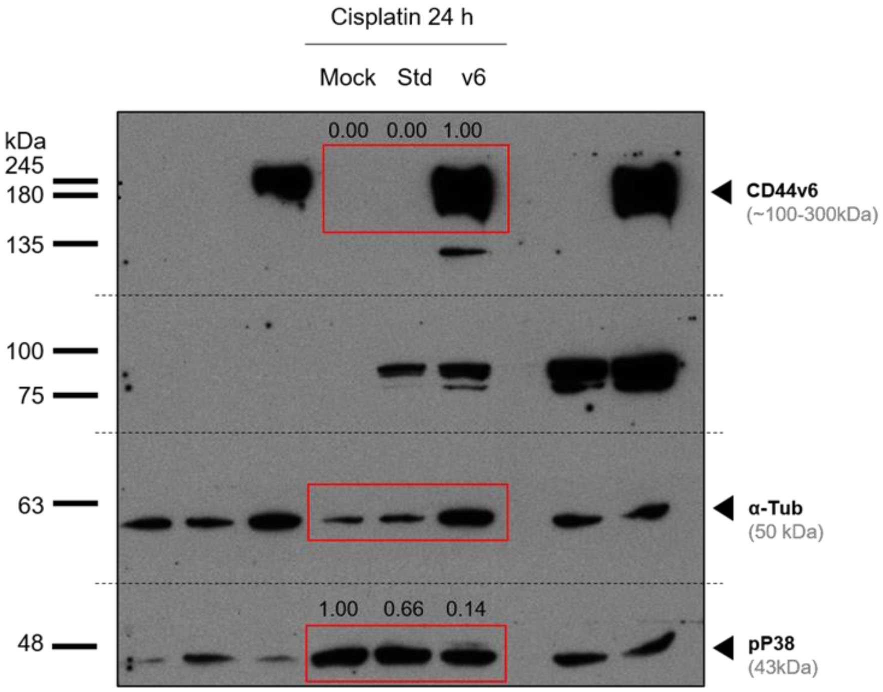

Figure S9. Original Western blots of Figure S4A.

Table S1. Primers used in the characterization of v6 containing transcripts in the GP202 cells.

Orientation	In-text Primer Name	Binding Site	Primer Sequence (5′ – 3′)	Melting Temperature	Comment
Forward	A	Exon 5	CATCCCAGACGAAGACAGTCC	Tm = 66.0 °C	
	B	Exon v6	CCCATTCGACAACAGGGACA	Tm = 69.1 °C	
	C	Exon 5/ v3	GAATCCCTGCTACCAGTACG	Tm = 61.1 °C	
Reverse	D	Exon v6	TTGGCGATATCCCTCATGCC	Tm = 68.3 °C	
	E	Exon 16/ 17	GTGTCCATCTGATTCAGATCCA	Tm = 63.9 °C	

	F	Exon 17/ 18	TGATCAGCCATTCTGGAATTTG	Tm = 65.9 °C	
	G	Exon 19	GAATCTCTTCAACTTCTTCGAC	Tm = 58.6 °C	Amplifies specifically the short tailed isoform
	H	Exon 20	TCTTCATGTCCACATTCTGC	Tm = 61.4 °C	Amplifies the long and short tailed isoforms

© 2020 by the authors. Licensee MDPI, Basel, Switzerland. This article is an open access article distributed under the terms and conditions of the Creative Commons Attribution (CC BY) license (http://creativecommons.org/licenses/by/4.0/).
